# Epidemiological characteristics of cryptococcal meningoencephalitis associated with *Cryptococcus neoformans* var. *grubii* from HIV-infected patients in Madagascar: A cross-sectional study

**DOI:** 10.1371/journal.pntd.0007984

**Published:** 2020-01-13

**Authors:** Rivonirina Andry Rakotoarivelo, Mihaja Raberahona, Tahinamandranto Rasamoelina, Andriamihaja Rabezanahary, Fetra Angelot Rakotomalala, Tiana Razafinambinintsoa, Thomas Bénet, Philippe Vanhems, Mamy Jean de Dieu Randria, Luisa Romanò, Massimo Cogliati, Muriel Cornet, Mala Rakoto Andrianarivelo

**Affiliations:** 1 Service des Maladies Infectieuses, Centre Hospitalier Universitaire Tambohobe, Faculté de Médecine, Université de Fianarantsoa, Fianarantsoa, Madagascar; 2 Service des Maladies Infectieuses, Centre Hospitalier Universitaire Joseph Raseta Befelatanana, Antananarivo, Madagascar; 3 Centre d’Infectiologie Charles Mérieux, Université d’Antananarivo, Antananarivo, Madagascar; 4 Service de Pneumologie, Centre Hospitalier Universitaire Analakininina, Toamasina, Madagascar; 5 Service d’Hygiène, Epidémiologie et Prévention, Hospices Civils de Lyon, Lyon, France; 6 Laboratoire des Pathogènes Emergents, Fondation Mérieux, Centre International de Recherche en Infectiologie, INSERM U1111, CNRS UMR5308, ENS de Lyon, UCBL1, Lyon, France; 7 Laboratorio di Micologia Medica, Dipartimento di Scienze Biomediche per la Salute, Universita degli Studi di Milano, Milano, Italy; 8 Université Grenoble Alpes, CNRS, CHU Grenoble Alpes, Grenoble I NP, TIMC-IMAG, Grenoble, France; Rutgers University, UNITED STATES

## Abstract

Cryptococcal meningoencephalitis (CM) remains the most prevalent invasive fungal infection worldwide. The main objective of this study was to describe the prevalence of CM and cryptococcal infection in HIV-infected patients in Madagascar. The secondary objectives were to assess the adjusted prevalence of CM according to clinical presentation and patient characteristics, to determine crude 90-day survival according to cryptococcal antigen (CrAg) status and CM, and to identify the genotypes of *Cryptococcus* clinical isolates. This cross-sectional study was carried out at two urban hospitals in Antananarivo (central highlands) and Toamasina (east coast) between November 2014 and December 2016. Consecutive HIV-infected adults presenting with CD4 cell counts ≤200/μl were enrolled. Lateral flow immunoassays of CrAg were performed on serum for all patients, and on cerebrospinal fluid for patients with CM symptoms. MALDI-ToF MS, ITS sequencing, and determinations of the molecular and mating types of the isolates were performed. Fluconazole is the only drug for CM treatment available in Madagascar. Patients were treated orally, with high doses (1200 mg/day) for 10–12 weeks and then with 200 mg/day. Minimum inhibitory concentrations were determined for amphotericin B, flucytosine, voriconazole and fluconazole in E-tests. Overall prevalence was 13.2% (95% CI 7.9–20.3) for cryptococcal infection and 10.9% (95% CI 6.1–17.5) for CM, among the 129 HIV-infected patients studied. The 90-day mortality rate was 58.8% (10/17) in CrAg-positive patients and 17.9% (20/112) in CrAg-negative patients (*p*<0.001). The 13 *Cryptococcus* strains obtained at baseline were all *Cryptococcus neoformans* var. *grubii*, genotypes VNI-αA (3 isolates), VNII-αA (4 isolates) or hybrid VNI/VNII-αAAα (6 isolates), suggesting high diversity. Two strains acquired fluconazole resistance after four and five months of exposure, respectively. The prevalence of cryptococcosis is high in Madagascar and this serious condition is life-threatening in HIV-infected patients. These findings will be used to raise the awareness of national authorities to strengthen the national HIV/AIDS control program.

## Author summary

Cryptococcal meningoencephalitis (CM) remains the most prevalent invasive fungal infection worldwide, with an estimated mortality of 70% in sub-Saharan Africa. In this cross-sectional study, we investigated the prevalence, clinical features, case-management and outcome of CM in HIV-infected patients. We also molecularly characterized the *Cryptococcus* isolates from these patients. The study was conducted in the main hospitals of two geographically distant cities, one located at Antananarivo in the central highlands and the other at Toamasina on the east coast. The prevalence of cryptococcal infection was higher than previously reported global prevalence values, and most of the patients developed CM. Classical signs of meningoencephalitis, including headache, fever and neck pain, were observed, as reported in previous studies. Ninety-day mortality (58.8%) was higher in patients with *Cryptococcus* infections than in non-infected patients (17.9%). All patients diagnosed with infection were treated with high-dose fluconazole, the only treatment available in Madagascar, in accordance with WHO recommendations. Molecular analyses of isolates from both regions revealed high levels of genome diversity and suggested that further environmental and larger clinical studies would be worthwhile. In conclusion, the prevalence of cryptococcal diseases is high in Madagascar, which is currently faced with the challenges of prioritizing various diseases of public health concern, providing health centers with rapid diagnostic tools and facilitating access to more reliable molecules.

## Introduction

Cryptococcal meningoencephalitis (CM) is a common opportunistic infection in developing countries, including those of sub-Saharan Africa. It mostly affects severely immunocompromised HIV-infected patients. With an estimated 625,000 deaths annually [1] and a global burden of almost a million cases per year [2], cryptococcal infection is one of the most devastating infectious diseases in sub-Saharan Africa and worldwide. A recent update of the global burden of HIV-associated cryptococcal meningoencephalitis (CM) reported an annual incidence of 162,500 cases in sub-Saharan Africa, accounting for 73% of the estimated number of cases globally and 135,500 deaths [3].

Most cryptococcal infections in HIV-infected patients are caused by the *Cryptococcus neoformans* (*Cn*) species complex, which includes two varieties and five molecular types: VNI, VNII and VNB (*Cn* var. *grubii* serotype A), VNIV (*Cn* var. *neoformans* serotype D), and VNIII, corresponding to intervarietal hybrids [4,5]. A recent taxonomic revision has also been proposed, in which *Cn* var. *neoformans* and *Cn* var. *grubii* are considered to be separate species [6]. VNI is the most prevalent molecular type of the *Cn* species complex worldwide; it is found in diverse environments, including bird guano, decaying wood, trees and soil [7], and can be transmitted to humans by basidiospore or small-sized blastospores inhalation, leading to primary lung colonization. Central nervous system invasion occurs principally in immunocompromised individuals, including people living with HIV [8], and results in CM, the most common and severe clinical form of cryptococcal infection [9].

The recent development of antigenic and molecular techniques has greatly improved the diagnosis of cryptococcal infection. The lateral flow immunoassay (LFA) has proved to be a sensitive point-of-care test capable of detecting the cryptococcal antigen (CrAg) rapidly, which should make it possible to implement treatment early, to reduce mortality [10].

With the spread of antiretroviral therapy (ART) in developed countries, the incidence of cryptococcal infection has greatly decreased. In developing countries, where access to ART is more limited, HIV infection remains the main risk factor for CM [11] and the case-fatality rate associated with CM can reach up to 70% in sub-Saharan Africa [1]. Little is known about the epidemiology, prevalence, associated clinical features and population diversity of the *Cryptococcus* species complex in Madagascar, in a context of low HIV/AIDS prevalence [12].

The main objective of this study was to evaluate the prevalence of CM and cryptococcal infection in HIV-infected patients. The secondary objectives were to assess the CM prevalence adjusted for clinical presentation and patient characteristics, to determine crude 90-day survival according to CrAg status and CM, and the genotypes of clinical isolates of *Cryptococcus*.

## Materials and methods

### Study design and population

This cross-sectional study was conducted at two urban hospitals: the Infectious Diseases department of Befelatanana University Hospital, Antananarivo, in the central highlands, and the Pneumology department of the University Hospital Analakininina, Toamasina, on the east coast. Consecutive HIV-infected adults presenting with CD4 cell counts ≤ 200/μl were enrolled between November 2014 and December 2016. As the primary objective was descriptive (i.e. estimation of the prevalence of cryptococcal meningoencephalitis in HIV positive individuals) and no previous data was available, we did not calculate sample size. Indeed, our cross-sectional study is the first estimate of the prevalence in Madagascar. Based on our result, with a sample size of 129 patients and the observed prevalence, the precision of the prevalence was lower than +/-7%. Study participants were not required to be ART naïve. Patients with a known history of cryptococcal disease were excluded from the study. A standardized case record form detailing sociodemographic characteristics, clinical signs and symptoms, was completed for each individual. The WHO recommends a one-week short-course induction regimen including amphotericin B deoxycholate (1 mg/kg/day) and flucytosine (100 mg/kg/day), followed by 1200 mg/day fluconazole for one week and then 800 mg/day fluconazole for eight weeks as a consolidation regimen, for the treatment of immunocompromised patients with CM [15]. However, amphotericin B and flucytosine are unavailable in Madagascar. Patients therefore received high-dose oral fluconazole alone (at least 1200 mg/day) for 10–12 weeks, followed by maintenance therapy with 200 mg/day fluconazole.

Ethics approval for the study was obtained from the National Ethics Committee of the Ministry of Health of Madagascar (authorization 111-MSANP/CE, dated 03/11/2014). Written informed consent was provided individually by all study participants, or by the close relatives of unconscious patients.

### Sample collection and laboratory testing

CD4 cell counts were determined with blood samples collected during routine immunological testing before the enrolment of the patient in the study. If CD4 cell count was below 200 cells/μl, the corresponding serum was tested for the presence of CrAg, by LFA, performed according to the manufacturer’s instructions (IMMY, Oklahoma, USA) [13]. If a positive result was obtained, the patient was hospitalized, and a detailed interview and clinical examination were performed to check for signs of meningoencephalitis (e.g., headache, meningism, fever, photophobia, or altered mental status). Patients with negative LFA results attended a check-up visit after six months. If meningoencephalitis was clinically suspected, lumbar puncture was performed to check for the presence of CrAg in the cerebrospinal fluid (CSF). CSF pressure was measured manually in a sterilized tube. For cases of confirmed infection, CrAg was quantified in CSF specimens at an initial dilution of 1:5, and then in two-fold serial dilutions to 1:1280, and a direct microscopic examination was performed after staining of the sample with India ink. HIV-1 RNA viral load (HIV VL) was quantified in plasma samples from study participants with the Generic HIV Viral Load assay (Biocentric, Bandol, France), as previously described [14]. The threshold for detection of this assay was 250 copies/ml (2.4 log_10_ copies/ml).

Other opportunistic infections were diagnosed based on clinical findings (consistent clinical presentation, duration of symptoms and therapeutic response to specific treatment), radiological findings, CD4 cells count, histological findings and confirmatory biological tests.

### Mycological and molecular analyses

Sabouraud dextrose agar (Biokar Diagnostics, Beauvais, France) tubes supplemented with chloramphenicol were inoculated with CSF samples from patients with clinically suspected CM, incubated at 30°C, and examined every two days. Genomic DNA was extracted with the GenElute Mammalian Genomic DNA Miniprep Kit (Sigma-Aldrich), and the internal transcribed spacers (ITS) region, including the 5.8S rRNA gene (514 bp), was sequenced as previously described [15]. The sequences were assembled and compared with sequences present in the International Society for Human and Animal Mycology (ISHAM) ITS Database [16]. The fungal isolates were then sent to Grenoble Alpes University Hospital in France for matrix-assisted laser desorption ionization time-of-flight (MALDI-ToF) mass spectrometry with a Bruker BioTyper instrument [17], and to Università degli Studi di Milano in Italy for additional molecular analyses. We determined the molecular and mating types allele patterns of the isolates, by performing two specific multiplex PCRs, as described elsewhere [18–20]. Strains H99 (VNI-αA), IUM 96–2828 (VNI-aA), WM626 (VNII-αA), JEC21 (VNIV-αD), JEC20 (VNIV-aD), and CBS132 (VNIII-αADa) were used as reference strains. The minimum inhibitory concentrations (MIC) for amphotericin B, flucytosine, voriconazole and fluconazole was determined with E-tests (Biomérieux, La Balme les Grottes, France), according to the manufacturer’s instructions. The MIC was determined after 72 h of incubation at 35°C on solid RPMI medium. In the absence of clinical breakpoints for the *Cryptococcus* species complex, we used the epidemiological cutoff values (ECV) established in previous studies [21,22]. Clinical Breakpoints are defined on the basis of five parameters: antifungal dose regimens, MIC distributions from multiple laboratories, ECV, pharmacokinetic/pharmacodynamics parameters and clinical outcome of multiple patients. ECV are defined with respect to the higher MIC of wild-type isolates.

### Statistical analysis

Categorical variables are expressed as numbers and percentages, with 95% confidence intervals (CI); continuous covariates are expressed as the median and interquartile range (IQR). Chi^2^ tests or Fisher’s exact tests were used to compare categorical variables. Continuous covariates were compared in Mann–Whitney *U*-tests or Kruskal–Wallis one-way analysis of variance. Univariate and multivariate Poisson regression analysis was performed, with robust modeling of variance error, to estimate the crude and adjusted prevalence ratios (PRs) of CM as a function of the principal characteristics of the patients. Patients without CM were used as the reference group. We preferred this type of modeling over logistic regression for the estimation of PRs [23]. Factors associated with patient survival were assessed with Kaplan–Meier curves and compared in log-rank tests. Follow-up and vital status recording were censored at 90 days after study enrolment for this analysis. Statistical analyses were performed with Epi Info 7.1.3 (Centers for Disease Control and Prevention, Atlanta, Georgia) and Stata 11.0 (StataCorp). All tests were two-tailed and a *p-*value of less than 0.05 was considered significant.

## Results

### Characteristics of the study population

In total, 129 HIV-infected patients were enrolled in this study (Table 1). The median age of participants was 37 years (IQR 32.0–45.0), and 62.0% were male. The median body mass index (BMI) was 17.6 kg/m^2^ (IQR 15.8–20.0). No differences were noted in age, sex or BMI between the 17 CrAg-positive (CrAg^+^) patients and the 112 CrAg-negative (CrAg^-^) patients. Overall, 58.9% of patients were inpatients, with a median duration of hospitalization of 22 days (IQR 11.0–35.0), and only 38.1% were prescribed ART. In total, 24.0% and 59.2% of the patients presented with disease at WHO clinical stages 3 and 4, respectively; the median baseline CD4 cell count was 83 cells/μl (IQR 31.5–123.5), and 62.8% of patients had a CD4 cell count <100 cells/ μl. The study participants included 88.1% with an HIV VL >250 copies/ml, the median HIV VL being 5.6 log_10_/ml (IQR 4.4–6.5). HIV VL did not differ significantly between CrAg^+^ and CrAg^-^ patients. Most patients (81.4%) had only recently been diagnosed with HIV infection. CrAg^+^ patients were more likely than CrAg^-^ patients to present low CD4 cell counts (*p* = 0.018).

**Table 1:** Descriptive analysis and characteristics of the HIV-infected patients at first visit with CD4 ≤ 200 cells/μl, Madagascar, 2014–2016.

Variable	Total	Antigenemia negative	Antigenemia positive	*p*
No. patients	129	112	17	
Sex, *n* (%)				
- Male	80 (62.0)	70 (62.5)	10 (58.8)	0.771
- Female	49 (38.0)	42 (37.5)	7 (41.2)	
Age, years, median (IQR)	37.0 (32–45)	37.0 (32–45)	37.0 (34–49)	0.531
BMI, kg/m^2^, median (IQR)	17.6 (15.8–20.0)	18.0 (16.0–20.3)	16.7 (12.8–18.0)	0.169
CD4 cell count, /μl, median (IQR)	83 (31.5–123.5)	86 (35–146)	38 (20–89)	0.018
CD4 cell count per μl, *n* (%)				
- < 100	81 (62.8)	65 (58.0)	16 (94.1)	0.017
- 100–200	48 (37.2)	47 (42.0)	1 (5.9)	
Median HIV-1 RNA, log_10_/ml (IQR), *n* = 126	5.6 (4.4–6.5)	5.5 (4.3–6.4)	6.0 (5.3–6.7)	0.085
HIV-1 VL interpretation, (%)				
- Detectable	111/126 (88.1)	96/110 (87.3)	15/16 (93.8)	0.454
- Undetectable	15/126 (11.9)	14/110 (12.7)	1/16 (6.3)	
WHO stage at enrolment, (%)				
- 1	10/125 (8.0)	10/108 (9.3)	0	0.242
- 2	11/125 (8.8)	10/108 (9.3)	1/17 (5.9)	
- 3	30/125 (24.0)	28/108 (25.9)	2/17 (11.8)	
- 4	74/125 (59.2)	60/108 (55.6)	14/17 (82.4)	
ART at baseline, *n* (%)				
- Yes	48/126 (38.1)	43/109 (39.4)	5/17 (29.4)	0.428
- No	78/126 (61.9)	66/109 (60.6)	12/17 (70.6)	
Hospitalization	76/129 (58.9)	63/112 (56.3)	13/17 (76.5)	0.114
Hospital stay, days, median (IQR)	22 (11.0–35.0)	23.0 (11.0–33.0)	21.0 (10.0–43.0)	0.923
Newly detected cases, n (%)	105/129 (81.4)	91/112 (81.3)	14/17 (82.4)	1.000
Manifestations, *n* (%)				
- Headache	44/125 (35.2)	29/108 (26.9)	15/17 (88.2)	<0.001
- Fever	88/127 (69.3)	74/110 (67.3)	14/17 (82.4)	0.21
- Neck pain	27/126 (21.4)	15/109 (13.8)	12/17 (70.6)	<0.001
- Night sweats	58/125 (46.4)	47/109 (43.1)	11/16 (68.8)	0.055
- Cough	78/128 (60.9)	68/111 (61.3)	10/17 (58.8)	0.848
- Photophobia	15/124 (12.1)	9/108 (8.3)	6/16 (37.5)	0.004
- Hearing disorders	13/125 (10.4)	8/108 (7.4)	5/17 (29.4)	0.017
- Blurred vision	20/124 (16.1)	17/108 (15.7)	3/16 (18.8)	0.722
Symptoms, *n* (%)				
- Temperature ≥38.0°C - Neck stiffness	43/126 (34.1) 16/126 (12.7)	35/109 (32.1) 8/109 (7.3)	8/17 (47.1) 8/17 (47.1)	0.751 <0.001
- Confusion	25/126 (19.8)	20/109 (24.5)	5/17 (29.4)	0.328
- Seizures	11/127 (8.7)	8/102 (7.3)	3/17 (17.7)	0.166
- Kernig’s sign	7/126 (5.6)	3/107 (2.7)	4/16 (25.0)	0.005
- Brudzinski’s sign	6/124 (4.8)	2/108 (1.9)	4/16 (25.0)	0.002
- Altered mental status	17/124 (13.7)	15/107 (14.0)	2/17 (11.8)	1
- Neurological deficit	14/127 (11.0)	14/110 (12.7)	0	0.213
Opportunistic infections, *n* (%)	67/104 (64.4)	61/92 (66.3)	6/12 (50.0)	0.339
- Pneumocystis pneumonia	47/103 (45.6)	41/91 (45.1)	6/17 (35.3)	0.746
- Tuberculosis	37/103 (35.9)	34/91 (37.4)	3/12 (25.0)	0.53
- Cerebral toxoplasmosis	15/103 (14.6)	12/91 (13.2)	3/12 (25.0)	0.376
- Kaposi’s sarcoma	5/104 (4.8)	5/92 (5.4)	0	1
- Other	12/104 (11.5)	11/92 (12.0)	1/12 (8.3)	1
Non OIs, *n* (%)	41/103 (39.8)	35/91 (38.5)	6/12 (50.0)	0.535
- Candidiasis	31/103 (30.1)	26/91 (28.6)	5/12 (41.7)	0.504
- Herpes	5/103 (4.9)	4/91 (4.4)	1/12 (8.3)	0.469
- Herpes zoster	4/103 (3.9)	4/91 (4.4)	0/12 (0)	1
- Pulmonary infection	11/103 (10.7)	10/91 (11.0)	1/12 (8.3)	1

OI: opportunistic infection IQR: interquartile range; VL: viral load; *p*: probability value

### Prevalence of cryptococcal infection and cryptococcal meningoencephalitis

Seventeen of the 129 HIV-infected patients enrolled had detectable CrAg in the blood; 14 of these patients also had CrAg in CSF and 13 had positive results for *Cryptococcus* spp. culture from CSF. The overall prevalence of cryptococcal infection was 13.2% (17/129, 95% CI 7.9–20.3), and that of CM was 10.9% (14/129, 95% CI 6.1–17.5). The 20.0% prevalence reported at the Toamasina site (6/30, 95% CI 7.7–38.6) was not significantly different from the 11.1% prevalence at Antananarivo site (11/99, 95% CI 5.7–19.1) [*p* = 0.21].

On stratification by CD4 cell count, 16 of the patients with CrAg in serum samples had CD4 cell counts ≤100 cells/μl (including the 14 patients with CrAg detection in CSF), whereas only one patient had a CD4 cell count between 100 and 200 cells/μl (*p* = 0.0029). Ten of the 14 CSF CrAg^+^ patients, 10 (71.4%) were severely immunocompromised, with CD4 cell counts <50 cells/μl (range: 4 to 44).

### Clinical features

At baseline, CrAg^+^ patients presented more frequently than CrAg^-^ patients with headache (*p*<0.001), neck pain (*p*<0.001), photophobia (*p* = 0.004), and hearing disorders (*p* = 0.017) (Table 1). On physical examination, CrAg^+^ patients had a higher prevalence of meningeal signs (neck stiffness, *p*<0.001; Kernig’s sign, *p* = 0.005; and Brudzinski’s sign, *p* = 0.002) than CrAg- patients. Amongst those with CM, there were 4 patients in whom ART was started before the diagnosis of CM (from 2 to 891 days). However, 2 of them have likely experienced a discontinuation of ART as their HIV viral load were detectable at inclusion. One patient has been prescribed ART 2 days before the diagnosis of CM and one patient, has been prescribed ART more than 2 years before the diagnosis of CM.

The following characteristics were associated with CM in robust univariate Poisson regression analysis: ART (crude prevalence ratio [cPR] = 0.3, 95% CI: 0.1–0.8), WHO category (cPR non estimable, *p*<0.001), CD4 cell count<100 cells/μl (cPR = 8.0, 95% CI: 1.1–59.5), neck pain (cPR = 22, 95% CI: 5.2–92.9), headache (cPR non estimable, *p*<0.001), hearing disorders (cPR = 3.4, 95% CI: 1.3–9.5), photophobia (cPR = 6.2, 95% CI: 2.4–16.1), night sweats (cPR = 4.9, 95% CI: 1.1–13.4), stiff neck (cPR = 6.9, 95% CI: 2.8–17.1), Kernig’s sign (cPR = 6.8, 95% CI: 2.8–16.3), Brudzinski’s sign (cPR = 7.9, 95% CI: 3.4–17.9), current shingles (cPR non estimable, *p*<0.001) and Kaposi’s sarcoma (cPR non estimable *p*<0.001). ART (adjusted prevalence ratio [PR] = 0.4, 95% CI: 0.2–0.9) and the presence of neck pain (adjusted PR = 15.7, 95% CI: 3.4–72.1) remained independently associated with CM after multivariate Poisson regression analysis adjusted for CD4 cell count.

CSF opening pressure data were available for seven of the 14 patients in whom CrAg was detected in the CSF; this pressure was high (>20 cm H_2_O) in three of these seven patients (42.9%; data not shown).

### Clinical outcome and analysis of risk factors

**Fig 1:**
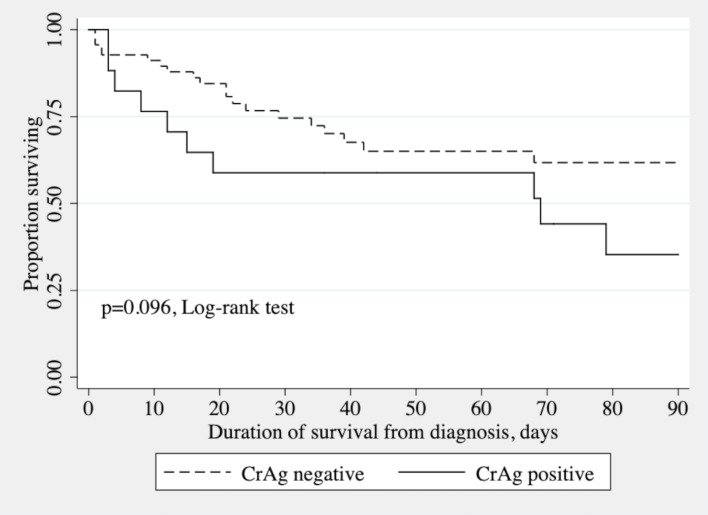
Kaplan-Meier survival curves for patients positive and negative for CrAg in tests on serum.

The 129 patients were hospitalized for 3648 patient-days in total, with 30 patients (23.3%) dying within 90 days. Amongst the 14 patients with CM, there were 9 deaths during the follow-up including 8 deaths occurring within 90 days. The outcome for one patient (CRY130) was unknown because he was lost to follow-up (Table 2). The 90-day mortality rate was 58.8% (10/17) for CrAg^+^ patients and 17.9% (20/112) for CrAg^-^ patients (*p*<0.001). Kaplan-Meier survival curves were plotted after stratification for CrAg status (Fig 1; *p* = 0.096, log-rank test). Survival at 30, 60 and 90 days was 75% (95% CI 61–84%), 65% (50–76%) and 62% (46–74%), respectively, for CrAg^-^ patients; and 59% (32–78%), 59% (32–78%), and 35% (12–59%), respectively, for CrAg^+^ patients. The 90-day mortality rate was 57.1% (8/14) in patients with CM and 19.1% (22/115) in patients without CM (*p* = 0.004). Survival at 30 days, 60 days and 90 days was 73% (95% CI 60–82%), 64% (49–75%) and 61% (46–73%), respectively, for patients without CM; and 64% (34–83%), 64% (34–83%), and 34% (10–61%), respectively, for patients with CM.

**Table 2:** Changes in cryptococcal antigen titers and viable yeasts in the CSF of the 14 patients with cryptococcal meningitis.

Case ID	CrAg titer					Culture	Outcome
	Day 1	Day 15/29	Day 30/59	Day 60/79	Day 90/120		
CRY017	≥1:1280					pos day 1	dead day 4
CRY033	1:20	1:10				neg day 11	dead day 69
CRY041	1:640	1:320	1:320	1:160	1:40	neg day 104	dead day 141
CRY061	≥1:1280					pos day 1	dead day 12
CRY067	1:320	1:640	1:40	1:40		neg day 39	dead day 68
CRY069	≥1:1280	1:1280	1:40	1:40		neg day 28	alive day 197
CRY070	≥1:1280	≥1:1280	≥1:1280	≥1:1280		pos day 56	dead day 73
CRY094	≥1:1280					pos day 1	dead day 19
CRY101	≥1:1280					pos day 1	dead day 4
CRY112	1:640	1:320	1:160		1:160	neg day 122	alive day 160
CRY114	≥1:1280					pos day 1	dead day 12
CRY124	1:320					na	alive day 120
CRY128	≥1:1280	1:80	1:40	1:40	1:40	neg day 77	alive day 120
CRY130	1:1280					pos day 1	lost to follow-up

na, not applicable (insufficient quantity)

Note: all patients were treated with fluconazole, except for patient CRY128, who received amphotericin B and high-dose fluconazole

### CrAg titration and culture of serial lumbar puncture specimens during treatment

Fourteen of the 17 CSF samples processed at baseline tested positive for CrAg and for India ink staining, with 100% concordance between the results obtained with these techniques (Table 2). In one case (CRY08124), too little CSF was available for culture. Yeasts grew from the remaining 13 samples on Sabouraud-chloramphenicol media, and the CrAg LFA test confirmed the diagnosis of CM in these cases.

CrAg titration results for the CSF are shown in Table 2. At baseline, nine patients had very high titers (≥1:1280), four had high titers (1:320–640), and one had a low titer (1:20). On follow-up, six patients with very high titers (≥1:1280) underwent only one CrAg titer determination due to premature death (CRY017, -061, 094, -101 and -114) or loss to follow-up (CRY130). One patient (CRY124) with a high titer (1:320) was not monitored but was reported to be alive 120 days post-treatment.

In the remaining seven patients, the clearance of viable yeasts was observed relatively early, at day 11, in one case (CRY033), and late (days 28 to 122) for the other six cases (CRY041, -067, -069, -070, -112 and -128), even on high-dose fluconazole. Fungal load remained persistently high in the CRY070 isolate. These finding suggest that the infection clearance rate is low.

Culture and India ink staining were systematically performed to assess the clearance of viable yeasts during patient monitoring. In total, 17 isolates were retrieved from the 13 patients for molecular analyses.

### Molecular identification and typing

All 17 clinical isolates obtained from CSF cultures (13 at baseline and 4 during follow-up) were identified as *Cn* var. *grubii* by ITS sequencing and MALDI-ToF mass spectrometry (Table 3). The 13 initial isolates were genotyped. They belonged to the most widely distributed VNI type (3 isolates), the rarer but also globally distributed VNII type (4 isolates) and a hybrid intravarietal type VNI/VNII (6 isolates). All three molecular types were found at Antananarivo, whereas only VNI/VNII was found at Toamasina. The molecular types VNI and VNII were mating-type αA, and molecular type VNI/VNII was mating-type αAAα.

**Table 3:** Molecular and mating types, and antifungal susceptibility of *Cryptococcus neoformans* var. *grubii* isolated from HIV-infected patients with cryptococcal meningitis in Madagascar.

Case ID	Date of isolation	Age/ Sex	Location	CD4 count (cell/μl)	MALDI-ToF	Molecular type	Mating type	Minimum inhibitory concentration (MIC, μg/ml)			
								AMB^a^ (ECV^e^ = 1)	5FC^b^ (ECV^e^ = 16)	VRZ^c^ (ECV^e^ = 0.5)	FLZ^d^ (ECV^e^ = 32)
CRY017	29/01/2015	34/F	Antananarivo	4	*C*. *neoformans* var. *grubii*	VNI	αA	0.190	>32	0.047	12.0
CRY033	17/04/2015	50/M	Antananarivo	89	*C*. *neoformans* var. *grubii*	VNII	αA	0.250	>32	0.016	1.0
CRY041	07/07/2015	38/M	Antananarivo	27	*C*. *neoformans* var. *grubii*	VNII	αA	0.064	6	0.047	12.0
	17/07/2015			nd	*C*. *neoformans* var. *grubii*	nd	nd	0.047	6	0.047	12.0
	04/08/2015			nd	*C*. *neoformans* var. *grubii*	nd	nd	0.500	>32	0.500	48.0
CRY061	10/08/2015	29/M	Toamasina	16	*C*. *neoformans* var. *grubii*	VNI/VNII	αAAα	0.250	>32	0.032	32.0
CRY067	29/08/2015	35/F	Toamasina	20	*C*. *neoformans* var. *grubii*	VNI/VNII	αAAα	0.190	>32	0.094	12.0
CRY069	14/09/2015	42/M	Toamasina	38	*C*. *neoformans* var. *grubii*	VNI/VNII	αAAα	0.125	>32	0.004	2.0
CRY070	21/09/2015	60/M	Antananarivo	19	*C*. *neoformans* var. *grubii*	VNI	αA	0.125	>32	0.064	12.0
	17/11/2015			nd	*C*. *neoformans* var. *grubii*	nd	nd	0.500	>32	0.064	12.0
CRY094	11/01/2016	48/M	Antananarivo	22	*C*. *neoformans* var. *grubii*	VNI/VNII	αAAα	0.032	8	0.047	12.0
CRY101	07/03/2016	36/F	Antananarivo	24	*C*. *neoformans* var. *grubii*	VNI	αA	0.064	4	0.064	8.0
CRY112	10/05/2016	50/M	Antananarivo	92	*C*. *neoformans* var. *grubii*	VNII	αA	0.250	4	0.016	2.0
CRY114	10/05/2016	42/F	Antananarivo	86	*C*. *neoformans* var. *grubii*	VNII	αA	0.320	>32	0.016	2.0
CRY128	10/10/2016	37/M	Antananarivo	19	*C*. *neoformans* var. *grubii*	VNI/VNII	αAAα	0.250	>32	0.064	12.0
	01/12/2016			nd	*C*. *neoformans* var. *grubii*	nd	nd	0.380	>32	0.064	>256
CRY130	08/12/2016	30/M	Toamasina	199	*C*. *neoformans* var. *grubii*	VNI/VNII	αAAα	0.190	>32	0.047	0.5

^a^ AMB, amphotericin B

^b^ 5FC, 5-flucytosine

^c^ VRZ, voriconazole

^d^ FLZ, fluconazole

^e^ ECV, epidemiological cut-off value (μg/ml)

nd, not done

### Antifungal susceptibility

All 17 *Cn* var. *grubii* isolates were tested (Table 3). Two isolates (CRY128 and CRY041) acquired fluconazole resistance after four and five months of exposure, respectively. Surprisingly, the MICs of two isolates from CRY070 case, described earlier and collected almost two months apart, were within the normal range, as defined by the ECV.

## Discussion

A high overall prevalence of CM (10.9%) and cryptococcal infection (13.2%) was found at the two hospitals located in the central highlands (Antananarivo) and on the east coast (Toamasina). The prevalence of cryptococcal infection in Toamasina (20.0%) did not differ significantly from that in Antananarivo (11.1%). After stratification by CD4 cell count, most cases of CrAg^+^ (94.1%) had <100 cells/μl. These findings are not surprising, because a high prevalence was expected in inpatients presenting with advanced HIV disease, as in our study, in which 83.2% of patients had reached WHO stage 3–4. By comparison, a mean global prevalence of cryptococcal infection of 6.0% was reported in outpatients with a CD4 cell count <100/μl from 29 countries [3]. The high prevalence observed here may also be explained by the small proportion of patients on ART before the development of CM. The high median viral load (5.6 log_10_/ml) was associated with a low median CD4 cell count (83 cells/μl), confirming the presence of advanced immunosuppression.

Immediately after diagnosis, patients with cryptococcal infection and CM were treated with at least 1200 mg fluconazole daily for at least 10 to 12 weeks, or until CSF cultures gave negative results for patients with CM. Outcome was poor in CrAg^+^ patients, who had a higher 90-day mortality rate (58.8%) than CrAg^-^ patients (17.9%). The causes of death were attributed to the following factors, either separately or in combination. First, most of the patients (81.4%) were diagnosed with HIV infection and cryptococcal disease at the same time, and were already severely immunosuppressed. Second, for the other patients, who received ART, poor compliance, as revealed by the high rates of virological failure, may have worsened HIV status, leading to opportunistic infections. Third, monotherapy with fluconazole, which is fungistatic rather than fungicidal, even at high doses, may not be optimal for the induction treatment of CM or cryptococcal infection [24–26], and should be replaced with the recommended regimen based on amphotericin or fluconazole combined with flucytosine [27]. Only one patient was treated with a combination of amphotericin and high-dose fluconazole, and this patient survived and was discharged.

Confirmatory tests for other opportunistic infections like pneumocystis pneumonia, cerebral toxoplasmosis and Kaposi sarcoma have been carried out on case-by-case basis and depending on whether or not the patient could bear the cost of these tests. Consequently, the burden of these opportunistic infections, especially pneumocystis pneumonia could potentially be overestimated. However, the presence of multiple opportunistic infections confirms the severe immunosuppression amongst these HIV-infected patients and seems to be a common situation in the study settings. The development of more reliable and convenient diagnostic tools has improved our knowledge of the infectious disease burden. This study provided us with an opportunity to set up and demonstrate the utility of the point-of-care CrAg LFA in the hospitals of Madagascar, as already described [28]. This study also facilitated the implementation of an open polyvalent platform using the French ANRS generic assay to measure HIV VL [14]. Our goal was to increase the capacity of the laboratory, and, subsequently, to improve access to HIV VL determinations in Madagascar. From August 2015 to December 2018, almost 1,951 HIV VL determinations from 1315 patients were available (Mala Rakoto Andrianarivelo, personal communication).

This study describes the first molecular characterization of *Cn* var. *grubii* isolates in Madagascar and reveals the presence of molecular type VNI, mating type αA (3 isolates), molecular type VNII mating type αA (4 isolates) and hybrid intravarietal VNI/VNII mating type αAAα (6 isolates). Molecular type VNI is the most prevalent worldwide, sub-Saharan Africa included [4]. Molecular type VNII is also widely distributed, but seems to be rare or underestimated [20]. Clinical and virulent hybrid intravarietal VNI/VNII isolates were isolated in the 1990s in Australia and Colombia [29], but the distribution of this molecular type remains poorly documented. In our study, CM associated with these three molecular types occurred in Antananarivo, in the central highlands, whereas only VNI/VNII was detected in the port city of Toamasina. Our findings suggest that *Cn* var. *grubii* displays considerable genetic diversity in both regions. The major environmental sources of the two varieties of *Cn* are soil contaminated with excreta from pigeons and other birds, and, less frequently, decaying wood and trees such as *Eucalyptus* [4,9], which are very common in Madagascar. It would be interesting to extend the clinical study to other representative regions of Madagascar and to conduct an environmental investigation to identify the ecological niche of *Cn* and to understand its impact on human populations.

The rapid acquisition of resistance to fluconazole, as observed in two isolates in this study, provides an additional argument for making amphotericin B and 5-fluorocytosine available. One of the serious difficulties encountered in the management of patients with cryptococcosis in Madagascar is the lack of alternatives to fluconazole. The purchase of amphotericin B and 5-fluorocytosine was initially planned, but the restrictive administrative procedures for importation, including an obligation to order much larger quantities of drugs than were required for this study, prevented us from making such an order.

In conclusion, this study highlights the high prevalence of cryptococcal infection and CM in HIV-infected patients with CD4 cell counts < 200/μl. CM is a major killer of HIV-infected patients in Madagascar. The LFA is a rapid test that is easy to perform and should be made available in HIV care centers for confirmation of the diagnosis. Monotherapy with oral fluconazole and the non-availability of other drugs against cryptococcosis remain crucial issues. The conclusions of this study will be used to raise the awareness of national authorities, to reinforce the national HIV/AIDS control program.

## Supporting information

S1 Checklist Strobe checklist. (DOCX)

S1 Appendix De-identified raw data. (XLSX)
